# Mechanisms by Which Interleukin-6 Attenuates Cell Invasion and Tumorigenesis in Human Bladder Carcinoma Cells

**DOI:** 10.1155/2013/791212

**Published:** 2013-05-16

**Authors:** Ke-Hung Tsui, Shyi-Wu Wang, Li-Chuan Chung, Tsui-Hsia Feng, Tzu-Yi Lee, Phei-Lang Chang, Horng-Heng Juang

**Affiliations:** ^1^Department of Urology, Chang Gung Memorial Hospital, Kwei-Shan, Taoyuan 33305, Taiwan; ^2^Bioinformation Center, Chang Gung Memorial Hospital, Kwei-Shan, Taoyuan 33305, Taiwan; ^3^Department of Physiology, Collage of Medicine, Chang Gung University, Kwei-Shan, Taoyuan 33302, Taiwan; ^4^Department of Anatomy, Collage of Medicine, Chang Gung University, Kwei-Shan, Taoyuan 33302, Taiwan; ^5^School of Nursing, Collage of Medicine, Chang Gung University, Kwei-Shan, Taoyuan 33302, Taiwan

## Abstract

Interleukin-6, a multifunctional cytokine, contributes to tumor cell proliferation and differentiation. However, the biological mechanisms that are affected by the expression of interleukin-6 in bladder cancer cells remain unclear. We evaluated the effects of interleukin-6 expression in human bladder carcinoma cells *in vitro* and *in vivo*. The results of interleukin-6-knockdown experiments in T24 cells and interleukin-6-overexpression experiments in HT1376 cells revealed that interleukin-6 reduced cell proliferation, migration, and invasion *in vitro*. Xenograft animal studies indicated that the overexpression of interleukin-6 downregulated tumorigenesis of bladder cells and that interleukin-6 knockdown reversed this effect. The results of RT-PCR, immunoblotting, and reporter assays indicated that the overexpression of interleukin-6 upregulated the expression of the mammary serine protease inhibitor (MASPIN), N-myc downstream gene 1 (NDRG1), and KAI1 proteins in HT1376 cells and that interleukin-6 knockdown reduced the expression of these proteins in T24 cells. In addition, results of immunoblotting assays revealed that interleukin-6 modulated epithelial-mesenchymal transitions by upregulating the expression of the E-cadherin, while downregulation N-cadherin and vimentin proteins. Our results suggest that the effects of interleukin-6 on the regulation of epithelial-mesenchymal transitions and the expressions of the MASPIN, NDRG1, and KAI1 genes attribute to the modulation of tumorigenesis in human bladder carcinoma cells.

## 1. Introduction

Interleukin-6 (IL6), a multifunctional cytokine, may contribute to tumor cell proliferation and differentiation [1]. Studies have suggested that IL6 is associated with a number of biological functions in bladder cancer, including cell proliferation, cell transformation, inflammation, and detrusor smooth muscle contractility [2–5]. In clinical studies, intravesical-Bacillus Calmette-Guerin (BCG) treatment has reduced bladder cancer recurrence rates by inducing a series of complex systemic humoral and cellular immune responses [6]. Prior studies have indicated that BCG increased IL6 production in human and murine bladder cancer cell lines by inducing G1 cell-cycle arrest while attenuating cell proliferation [7, 8], and IL6 inhibited tumor growth in murine bladder transitional carcinoma *in vivo* [2]. However, the mechanisms by which IL6 affects human bladder carcinoma cells remain unclear.

The mammary serine protease inhibitor (MASPIN) is a member of the serine protease inhibitor superfamily and has been characterized as a class II tumor suppressor gene based on its ability to promote apoptosis and inhibit both cell invasion and angiogenesis [9]. In human bladder cells, the expression of MASPIN is correlated with the tumor invasiveness, angiogenesis, cell migration, cell dissemination, and inflammation [10–12]. The KAI1 gene, also known as CD82, is a membrane of the tetraspanin superfamily and is regarded as a metastasis-suppressor gene in several types of cancers [13]. Both *in vivo* and *in vitro* studies have shown that the downregulation of KAI1 transcription is associated with invasive bladder cancer and suggested that the KAI1 gene may function as an invasion/metastasis suppressor gene in bladder cancer [14, 15]. N-myc downstream regulated gene 1 (NDRG1) belongs to the NDRG family, and its expression has been shown to be negatively correlated with tumor metastasis [16]. The functions and regulatory mechanisms of NDRG1 gene have not been conclusively evaluated in human bladder carcinoma cells.

The epithelial to mesenchymal transition (EMT) plays a crucial role in the differentiation of multiple tissues and organs during embryogenesis [17]. Previous studies have concluded that EMT is associated with (1) cancer cell survival and resistance to apoptosis, (2) invasion and tumor angiogenesis, (3) metastasis and drug resistance of advanced tumors, and (4) tumorigenesis [18, 19]. Defining features of EMT in cancer are a reduction in E-cadherin levels and the concomitant production of N-cadherin [20]. Both the loss of E-cadherin expression and the gain of N-cadherin expression are important markers in bladder cancer progression [21].

Objectives of this study were to determine the effects of IL6 expression on cell proliferation, invasion, and tumorigenesis in bladder carcinoma cells *in vitro* and *in vivo*. The effects of IL6 on the dysregulation of EMT markers and the expressions of MASPIN, NDRG1, and KAI1 genes in bladder carcinoma cells were examined. 

## 2. Materials and Methods

### 2.1. Cell Culture and Chemicals

 The RT-4, HT1376, and T24 cells were obtained from Bioresource Collection and Research Center (BCRC, Taiwan). Fetal calf serum (FCS) was purchased from HyClone (Logan, UT, USA), and RPMI 1640 media were purchased from Life Technologies (Rockville, MD, USA). Matrigel was purchased from Becton Dickenson Biosciences (Bedford, MA, USA). 

### 2.2. Expression Vector Constructs and Stable Transfection

 The human IL6 expression vector was constructed as described previously [22]. 1 × 10^7^ HT1376 cells were combined with 20 *μ*g of the IL6 expression vectors in 500 *μ*L of serum-free RPMI medium in a 4 mm gap cuvette. Electroporation was performed using the ECM 830 (BTX, San Diego, CA, USA), with a singles 70 msec pulse of 190 V. Cells were maintained in an RPMI medium with 10% FCS and 100 *μ*g/mL Zeocin (Invitrogen, Carlsbad, CA, USA). The selected colonies were designated HT-IL6. The mock-transfected HT1376 cells (HT-DNA) were produced by transfection with the pcDNA3.1/Zeo control plasmid, and Zeocin-resistant clones were selected. The pSMc2 retroviral vectors containing the IL6 short hairpin RNA (shRNA; V2HS-111640) and the GFP shRNA (RHS1764-9394112) were purchased from Open Biosystems (Huntsville, AL, USA). The IL6 and GFP knockdown vectors were introduced into T24 cells by electroporation using a singles 70-msec pulse of 180 V, and the transfections were selected using 2 *μ*g/mL puromycin dihydrochloride. The IL6-knockdown T24 cells were designated T24-IL6si cells and GFP-knockdown T24 cells were designated T24-GFPsi cells. The expression of the IL6 gene in resistant colonies was evaluated by reverse transcription-polymerase chain reaction (RT-PCR) and enzyme-linked immunosorbent assay (ELISA), as described below.

### 2.3. Reverse Transcription-Polymerase Chain Reaction and Quantitative Real-Time Polymerase Chain Reaction

Total RNA was isolated using the TRIzol reagent, and cDNA was synthesized using the Superscript III preamplification system (Invitrogen). Primers V1 and V2 (5′-CTAACCAACGACAAAGCCC-3′ and 5′-GCTGTTCCTGAATCTGAGCC-3′) were used for the amplification of sequences specific to human vimentin mRNA. The sequence of the primers used to amplify sequences specific to the human *β*-actin and IL6 cDNA have been described previously [22]. The qPCR assays were performed using an ABI StepOne Plus Real-Time PCR system (Applied Biosystems, Foster City, CA, USA), as described previously [23]. FAM dye-labeled TaqMan MGB probes and PCR primers for human NDRG1 (Hs00608387_m1), MASPIN (Hs00985283_m1), and KAI1(Hs00356310_m1) were purchased from Applied Biosystems. The 18S ribosomal RNA primers (18S; Hs03003631_g1) were used as the internal positive controls. 

### 2.4. Immunoblot Assay

 Equal quantities of cell extracts were analyzed on a 10% acrylamide (w/v) using SDS-PAGE, and the Western lightning plus-ECL detection system (PerkinElmer, Inc., Waltham, MA, USA). The membranes were probed using following dilution of antibodies: 1 : 1000 anti-MASPIN (cat. no. 554292; BD Biosciences), 1 : 200 anti-KAI1 (G-2; Santa Cruz Biotechnology, Santa Cruz, CA, USA), 1 : 1000 anit-NDRG1 (cat. no. 42-6200; Invitrogen), 1 : 100 anti-E-cadherin (1.B.54; Santa Cruz Biotechnology), 1 : 1000 anti-N-cadherin (AJ1526a; Abgent, San Diego, CA, USA), 1 : 500 anti-vimentin (AJ1815a; Abgent), or 1 : 1000 anti-*β*-actin (I–19; Santa Cruz Biotechnology). The immunoblotting results were quantified using a ChemicGenius II BioImaging system (Syngene; Cambridge, UK).

### 2.5. Enzyme Linked Immunosorbent Assay

 The IL6 protein level in the culture medium or blood samples was measured by ELISA (cat. no. 2107; Bio Scientific Corporation, Austin, TX, USA), as described previously [24]. The relative mass of the IL6 protein present in each sample was determined based on the total protein concentration of the whole cell extract, as assessed using a bicinchoninic acid protein assay kit (Pierce Biotechnology, Rockford, IL, USA).

### 2.6. Cell Proliferation Assay

 The cellular proliferation of bladder carcinoma cells was measured using a ^3^H-thymidine incorporation assay, as described previously [25]. In brief, 5 × 10^4^ cells were cultured in 6-well plates in RPMI 1640 medium with 10% FCS, and 1 *μ*Ci/mL of ^3^H-thymidine (PerkinElmer, Bosto, MA, USA) was added. The cells were incubated at 37°C in a humidified atmosphere with 5% CO_2_ for 4 h. Cells were washed twice with cold PBS and once with cold 5% trichloroacetic acid. Cells were solubilized by adding 0.5 mL of 0.5N NaOH, and 400 *μ*L of the solubilized cell solution was combined with 4 mL scintillation cocktail for analysis using a Liquid Scintillation Analyzer (Packard BioScience, IL, USA). 

### 2.7. Matrigel Invasion Assay and Migration Assay

 The matrigel invasion assay was performed as described previously [26]. In brief, 500 *μ*L of RPMI1640 media with 10% FCS was added to the lower chamber of the 24-well plate. Culturing was performed using 200 *μ*L cells in serum-free RPMI1640 medium in the upper chamber at a cell density of 1 × 10^5^ cells/mL. The cells were cultured in 5% CO_2_ at 37°C for 24 h for T24 cells or 48 h for HT1376 cells. Cells that migrated to the opposite side of the matrigel-coated transmembrane were fixed in 4% paraformaldehyde and stained with 0.1% crystal violet for 30 min. The results were digitally recorded using an inverted microscope (IX71, Olympus, Tokyo, Japan). The membranes were soaked in 10% acetate acid and agitated at 37°C for 1 h. The acetate acid solutions were analyzed using a DU640 spectrophotometer at 635 nm (Beckman Coulter, Fullerton, CA, USA). The migration assay was performed using Costar transwell plates (cat. no. 3422, Corning, NY, USA) as described without the matrigel coating of the transmembrane.

### 2.8. Xenograft Tumors of T24 Cells in Nude Mice

These studies were performed in accordance with the Guide for Laboratory Animal facilities and Care as promulgated by Council of Agriculture Executive Yuan, Taiwan. The protocol was approved by the Chang Gung University Animal Research Committee (permit number: CGU08-74). Male nude mice (BALB/cAnN-Foxn1, 4 weeks old) were purchased from the animal center of the National Science Council in Taiwan. Before treatment, the mice were anesthetized intraperitoneally with ketamine (5 *μ*g/kg)-xylazine (0.5 *μ*g/kg). Equal volumes of T24 cells and matrigel were combined to enhance the tumorigenic activity of the cells, before a single anterior-dorsolateral subcutaneously inoculation consisting of 100 *μ*L of the mixture containing 1 × 10^6^ cells was administered to each mouse. All efforts were made to minimize suffering and performed. Growth of the xenografts was measured using vernier calipers at 5-day intervals. Tumor volume was calculated as *π*/6 × larger diameter × (smaller diameter)^2^ as described previously [26].

### 2.9. Reporter Vector Constructs and Transient Gene Expression Assay

 The reporter vectors for the MASPIN and NDRG1 genes were constructed as described previously [27]. The DNA fragment containing the 5′-flanking region of the MASPIN gene (−5948 to −5) and the promoter of the NDRG1 gene (−4714 to +46) were excised from commercially available BAC clone (RP11-851B10 and CTC-458A3, respectively; Invitrogen). A 5.9-kb DNA fragment of the KAI1 gene was excised from the BAC clone RP11-58 K22 (Invitrogen) and blunt-end ligated into the pGEM-5 vector (Promega BioScience, Madison, WI, USA) using the Eco RV restriction site. The DNA fragment containing the promoter and the 5′-flanking region of the KAI1 gene (−4343 to −1) was synthesized by PCR amplification using KAI1promP (5′-GGTACCTTCTCTCAAGGCTTTCTAGGG-3′) and KAI1promR (5′-AGATCTCCGGGGCTCAGTCACTCCTCGG-3′) oligonucleotide primers and ligated into the pGL3-Basic vector (Promega BioScience) using the Kpn I and Bgl II restriction sites. Cells were transiently transfected using the TransFast transfection reagent, as previously described [28]. The luciferase activity was adjusted for efficient transfection using the normalization control plasmid pCMVSPORT*β*gal.

### 2.10. Statistical Analysis

Results are expressed as mean ± standard error (SE) of at least 3 independent experiments. Statistical significance was determined by Student's *t*-test and one-way ANOVA using the SigmaStat program for Windows, version 2.03 (SPSS Inc., Chicago, IL, USA).

## 3. Results

### 3.1. Expression of IL6 in Bladder Carcinoma Cells

**Figure 1:**
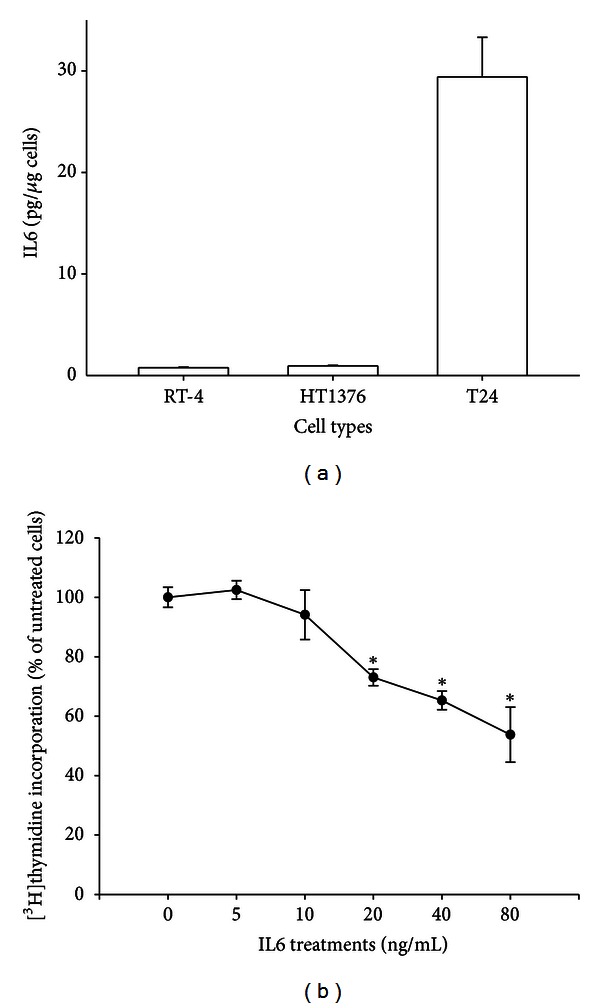
Effects of IL6 on cell proliferation in human bladder carcinoma cells. (a) Culture medium samples were collected for ELISA analysis to determine IL6 protein expression in different bladder carcinoma cell lines. Data are presented as mean (±SE, *N* = 6) of the IL6 protein levels. (b) HT1376 cells were treated with various concentrations of exogenous recombinant human IL6 as indicated for 72 h. Cell proliferation was determined using the ^3^H-thymidine incorporation method. Each point on the curve represents the mean percentage ±SE (*N* = 6) of ^3^H-thymidine incorporation in the control group. *P < 0.05.

 Results of ELISA analysis showed that RT-4 and HT1376 cells expressed significantly lower levels of IL6 compared with the T24 cells (Figure 1(a)). The IL6 expression levels in the RT-4, HT1376, and T24 cells were 0.76 ± 0.03, 1.00 ± 0.06, and 29.41 ± 3.90 pg/*μ*g cells, respectively. 

### 3.2. IL6 Exhibits Antiproliferation, Antimigration, and Antiinvasiveness on Bladder Carcinoma Cells *In Vitro*

**Figure 2:**
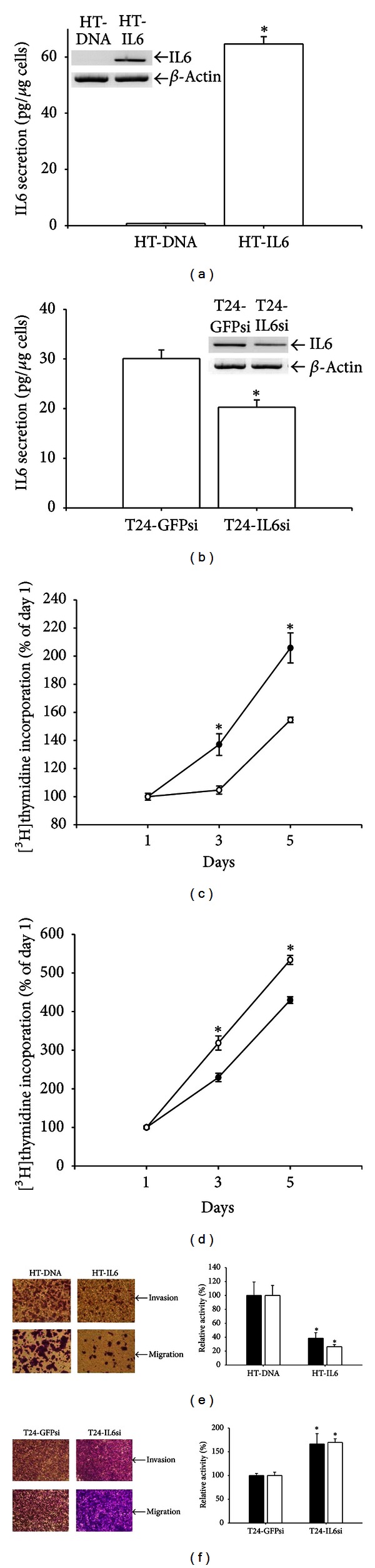
Effect of overexpression of IL6 and IL6knockdown on cell proliferation, migration, and invasion. The expression of IL6 in HT1376 cells was determined by RT-PCR and ELISA after stable transfection with the IL6 expression vector (a) and in T24 cells after IL6 knockdown treatment (b). Data are expressed as mean (±SE; *N* = 6) of the IL6 levels. (c) Cell proliferation of mock-transfected HT1376 cells (HT-DNA; black circle) and of IL6-transfected HT1376 cells (HT-IL6, white circle). (d) Cell proliferation of mock-IL6-knockdown T24 cells (T24-GFPsi; black circle) and of IL6-knockdown T24 cells (T24-IL6si, white circle). Cell proliferation was determined using the ^3^H-thymidine incorporation method. Each point on the curve represents the mean percent ±SE (*N* = 6) of ^3^H-thymidine incorporation on day 1. ∗ indicated a statistically significant difference of cell numbers relative to mock-transfected cells on the same day. Comparisons of the migration (white bars) and invasion (black bars) activities between the HT-DNA and the HT-IL6 cells (e) and between the T24-GFPsi and the T24-IL6si cells (f) were determined by *in vitro* invasion and migration assays. Experimental data are presented as the mean percentage ±SE (*N* = 3) of the absorbance in relation to that of the mock-transfected cell group. *P < 0.05.

Results of ^3^H-thymidine incorporation assay revealed that the inhibition of HT1376 cell growth occurred initially with 72 h treatments of 20 ng/mL of IL6, and the magnitude of inhibition increased in a dose-dependent manner. Seventy-two hours of 80 ng/mL IL6 treatments reduced cell proliferation by 46% (Figure 1(b)). The expressions of IL6 in the selected clones were determined by RT-PCR and ELISA analyses (Figures 2(a) and 2(b)). Our *in vitro* ^3^H-thymidine incorporation experiments revealed a 2.2-fold increase in HT-DNA cells over a 5-day interval. By contrast, the HT-IL6 cells increased 1.5-fold over the same period (Figure 2(c)). Results of ^3^H-thymidine incorporation assay also revealed a 4.1-fold increase in T24-GFPsi cells over 5-day interval and an increase in T24-IL6si cells of approximately 5.5-fold over the same period (Figure 2(d)). Results of *in vitro* invasion and migration assays showed that the invasion and migration of the HT-IL6 cells decreased by approximately 60% and 70%, respectively, as compared with the HT-DNA control cells (Figure 2(e)). Conversely, the invasion and migration of T24-IL6si cells increased 1.68- and 1.72-folds, respectively, compared with the T24-GFPsi cells (Figure 2(f)). 

### 3.3. IL6 Upregulates the Expression of NDRG1, MASPIN, and KAI1

**Figure 3:**
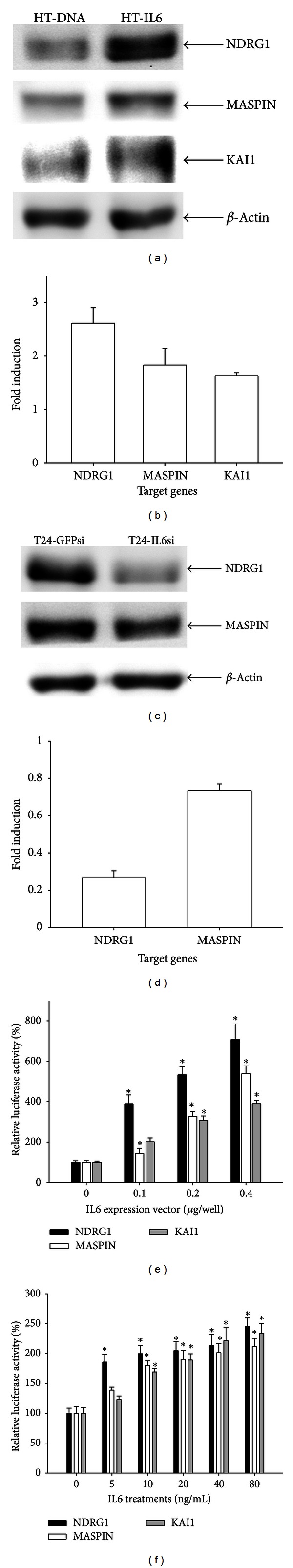
Expression of IL6 modulates NDRG1, MASPIN, and KAI1 gene expression in bladder carcinoma cells. (a) The expression profiles of the NDRG1, MASPIN, and KAI1 proteins in IL6-transfected cells (HT-IL6) and mock-transfected control cells (HT-DNA) were determined by immunoblotting analysis. (c) The expression profiles of the NDRG1 and MASPIN proteins in IL6-knockdown T24 cells and mock-IL6-knockdown T24 cells (T24-GFPsi) were determined by immunoblotting analysis. Quantitative analysis was based on the intensity of the protein bands produced by the expression of the target genes and the *β*-actin internal control gene in 3 independent experiments (b, d). The fold-induction data are presented as the band intensity corresponding to the target gene/*β*-actin gene (±SE) relative to the corresponding band intensity for the mock-treated cells. (e) The NDRG1 (black bars), MASPIN (white bars), and the KAI1 (gray bars) reporter vector-transfected HT1376 cells were cotransfected with different concentrations of IL6 expression vector for 72 h. (f) The NDRG1 (black bars), MASPIN (white bars), and the KAI1 (gray bars) reporter vector-transfected HT1376 cells were treated with different concentrations of exogenous recombinant human IL6 for 48 h. Cells were lysed for the luciferase assays. Data are presented as mean percentage ±SE. (*N* = 6) relative to the control groups. *P < 0.05.

 Results of immunoblot assays revealed that overexpression of IL6 increased the expression of the NDRG1, MASPIN, and KAI1 protein in HT1376 cells, based on the quantitative analysis of SDS-PAGE band intensities in 4 independent experiments (Figures 3(a) and 3(b)). By contrast, IL6 knockdown reduced the levels of the NDRG1 and MASPIN protein in T24 cells, as compared with the mock-knockdown T24 (T24-GFPsi) cells (Figure 3(c)). However, the KAI1 protein levels in both the T24-GFPsi and T24-IL6si cells were below detectable levels determining by immunoblotting assay (data not shown). The results of quantitative analysis are presented in Figure 3(d). The transient gene expression assays indicated that IL6 expression enhance luciferase activities from reporter vectors that used the 5′-flanking fragments of NDRG1, MASPIN, and KAI1 genes (Figure 3(e)). Results of the transient gene expression assays also indicated that treatment with exogenous recombinant human IL6 also increased the activity of the NDRG1, MASPIN, and KAI1 promoters (Figure 3(f)).

### 3.4. IL6 Modulates Protein Expression of E-Cadherin, N-Cadherin, and Vimentin in Bladder Carcinoma Cells

**Figure 4:**
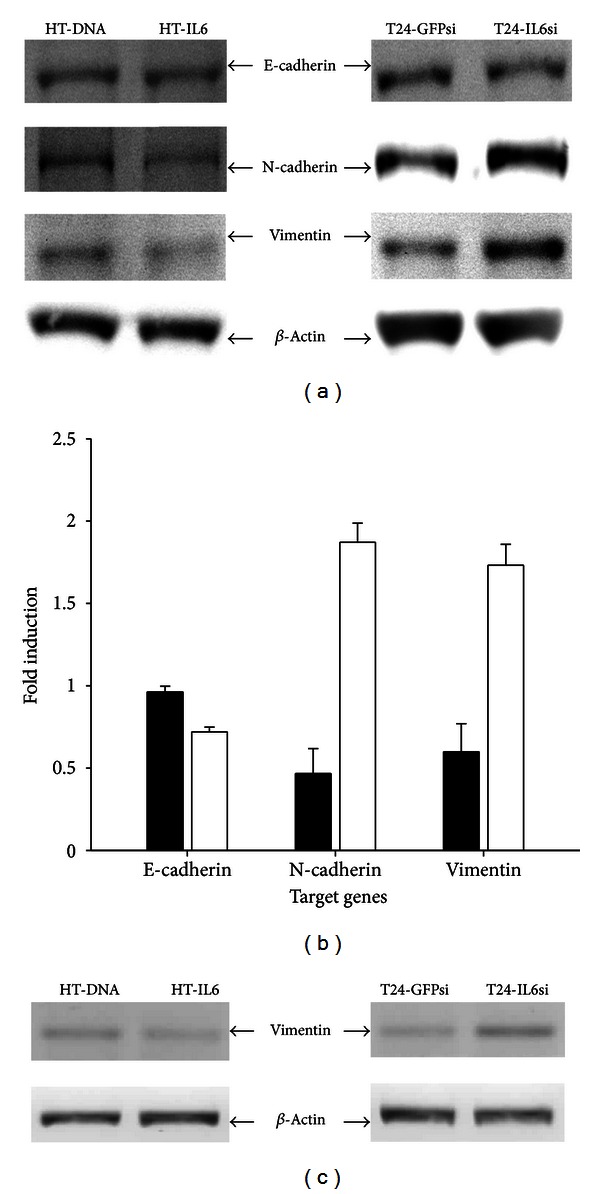
IL6 modulates the expression of E-cadherin, N-cadherin, and vimentin in bladder carcinoma cells. (a) The different levels of expression of E-cadherin, N-cadherin, and vimentin between HT-DNA and HT-IL6 cells (left) and between T24-GFPsi and T24-IL6si cells (right) were determined by immunoblotting analysis. (b) The quantitative analysis of HT-IL6/HT-DNA cells (black bars) and T24-IL6si/T24-GFPsi cells (white bars) was based on the intensity of the protein bands produced by the expression of the target genes and the *β*-actin internal control gene in 3 independent experiments. The fold-induction data are expressed as of the intensity of the protein bands produced from the target gene/*β*-actin (±SE) relative to that of the mock-treated cells. (c) The different levels of expression of vimentin between HT-DNA and HT-IL6 cells (left) and between T24-GFPsi and T24-IL6si cells (right) were determined by RT-PCR analysis.

 We compared the expression of E-cadherin, N-cadherin, and vimentin proteins in HT-IL6 and mock-transfected HT-DNA cells. Stable overexpression of IL6 in HT1376 cells did not affect the levels of E-cadherin protein but significantly reduced the levels of N-cadherin and vimentin proteins (Figure 4(a), left). Conversely, the results of immunoblot assays indicated that the levels of E-cadherin protein decreased while the levels of N-cadherin and vimentin increased in response to IL6 knockdown in T24 cells (Figure 4(a), right). The results of quantitative analysis are presented in Figure 4(b). The results of RT-PCR indicated that the expression of vimentin decreased in response to IL6 overexpression in HT1376 cells while it increased in response to IL6 knockdown in T24 cells (Figure 4(c)).

### 3.5. IL6 Exerts Antitumorigenic Activity in Bladder Carcinoma Cells *In Vivo*

**Figure 5:**
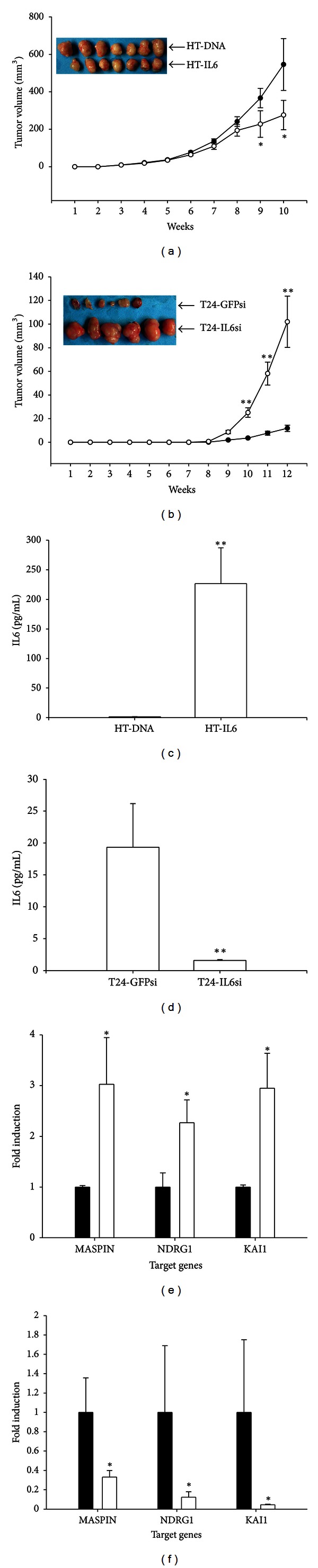
IL6 attenuates tumorigenesis of bladder carcinoma cells. (a) Nude mice were inoculated subcutaneously with HT-DNA (black circle) or HT-IL6 (white circle) cells. (b) Nude mice were inoculated subcutaneously with T24-GFPsi (black circle) or T24-IL6si (white circle) cells. Tumor size was measured with vernier calipers and the data are presented as tumor size in mm^3^ (±SE) at indicated time intervals (*P < 0.05). Blood samples were collected from experimental animals by cardiocentesis and were assayed for IL6 (c, d) levels by ELISA. Data are presented as the mean (±SE) of the IL6 protein levels. The expression of the MASPIN, NDRG1, and KAI1 genes in the tumor tissues (*N* = 3) of (e) the HT-DNA (black bars) and HT-IL6 (white bars) xenograft groups and (f) the T24-GFPsi (black bars) and the T24-IL6si (white bars) xenograft groups was determined using RT-qPCR. Data are presented as mean fold induction of the mRNA levels of the target gene (±SE) relative to the mock-transfected xenograft groups (*P < 0.05,  **P < 0.01).

The effect of IL6 on the growth of tumors *in vivo* was evaluated using xenograft in nude mice. The HT-IL6 cells generated tumors that grew at slower rate, as compared with the tumors produced from xenografts using mock-transfected HT-DNA cells. After 10 weeks of growth, tumors derived from HT-IL6 cells were approximately 50% of the size of the tumors produced using HT-DNA cells (Figure 5(a)). ELISA results from the analysis of serum samples that were collected by cardiocentesis showed that blood levels of IL6 were 200-fold higher in animals that had been injected with the HT-IL6 cells than animals that had been injected with HT-DNA cells (Figure 5(c)). The RT-qPCR analyses showed that expression from NDRG1, MASPIN, and KAI1 genes was significantly increased in the HT-IL6 xenograft tumor cells compared with HT-DNA xenograft tumor cells (Figure 5(e)). Conversely, tumor derived from mock-IL6-knockdown T24-GFPsi cells was approximately 10% of the average size of tumors derived from the IL6*-*knockdown T24-IL6si cells after 12 weeks of growth (Figure 5(b)). ELISA analysis of serum samples revealed that the blood levels of IL6 were 10-fold higher in animals that had been injected with the T24-GFPsi cells, compared with animals that had been injected with the T24-IL6si cells (Figure 5(d)). The RT-qPCR analyses showed that expression from NDRG1, MASPIN, and KAI1 genes was significantly decreased in the T24-IL6si cell-produced xenograft tumors as compared to those tumors produced by using T24-GFPsi cells (Figure 5(f)).

## 4. Discussion

Although studies found that plasma and urine IL6 levels were higher in patients with bladder cancer than in healthy controls [29, 30], unlike prostate cancer, the function of IL6 in bladder cancer remains unclear. Because IL6 plays a fundamental role in supporting the systemic host response to tissue injury, the higher levels of IL6 in bladder cancer patients may be the results of a complex systemic immune response mediated by peripheral blood mononuclear cells [31]. Other studies have indicated that IL6 attenuated cell proliferation of murine bladder transitional carcinoma cells* in vivo* and *in vitro* [2, 8]. However, an evaluation of the direct effects of IL-6 on human bladder carcinoma cells has not been well defined. 

The RT4 cell line is a p53 wild-type transitional-cell papilloma [32]. HT1376 cells are well-differentiated bladder-carcinoma cells with tumorigenic activity that were derived from a Grade 3 bladder carcinoma [33]. T24 cells are poorly differentiated bladder-carcinoma cells with no tumorigenic activity that were isolated from a transitional-cell carcinoma tumor [34]. Our results showed that the T24 cells produced higher levels of IL6, compared with the RT-4 and HT1376 cells, which is consistent with previous studies that indicated constitutive IL6 expression to be cell line dependent [29, 35].

Studies have shown that IL6 treatment enhanced cell proliferation in PC-3 and DU145 androgen-independent prostate-carcinoma cells lines [36]. However, another study indicated that IL6 causes growth arrest and induces differentiation of the LNCaP androgen-sensitive human prostate-carcinoma cell line [22]. Moreover, an earlier previous study showed that IL6 treatment increased the proliferation of LNCaP-IL6+ cells, a subline of LNCaP cells established by long-term IL6 treatment, suggesting that the effect of IL6 on cell proliferation *in vitro* is cell and microenvironment specific in prostate carcinoma cells [37]. The results of IL6 overexpression and the exogenous IL6 treatment in our study showed that IL6 attenuated HT1376 cell proliferation. Similar results have also been found in a study of murine bladder transitional carcinoma [2]. 

Results of our xenograft animal study show that the overexpression of IL6 attenuated tumorigenesis in HT1376 cells and that IL6 knockdown enhanced tumorigenesis in T24 cells. The mechanism by which IL6 affects the tumorigenicity of these bladder carcinoma cell lines remains unclear. The results of a similar xenograft animal study using the BTT739 murine bladder transitional carcinoma cell line indicated that recombinant human IL6 blocked tumorigenesis through Fas-mediated apoptosis [2]. Collectively, the results of our ^3^H-thymidine incorporation experiments and our xenograft animal studies indicated that IL6 attenuates the proliferation of human bladder carcinoma cells *in vitro* and *in vivo*. 

Our results showed that IL6 overexpression upregulated the activity of the MASPIN promoter in HT1376 cells and that IL6 knockdown downregulated MASPIN expression at the transcriptional level in T24 cells. These results are contrary to observations of the IL6-dependent downregulation of the MASPIN in PC-3 prostate carcinoma cells [36]. However, the divergent effects of IL6 on the expression of MASPIN are consistent with the contrary effect of IL6 on cell proliferation and invasion in bladder carcinoma, compared with the effects of IL6 on these aspects of tumorigenesis in prostate carcinoma that were also reported in Santer et al. [36].

NDRG1 is involved in cellular differentiation, proliferation, and growth arrest, as well as neoplasia, tumor progression and metastasis, heavy metal responses, the hypoxia response, and DNA damage response [38]. Reports have indicated that NDRG1 inhibits tumor growth and metastasis in several types of cancer [23, 24, 39]. However, the role of NDRG1 in bladder cancer has remained unclear. The results of our study indicate that IL6 upregulated the expression of NDRG1 in bladder carcinoma cells.

KAI1 has been shown to function as a metastasis suppressor gene in bladder cancer [14, 15, 40]. However, a regulatory mechanism for the expression of KAI1 in bladder cancer has not been identified. Our study represents the first report showing that IL6 upregulates the expression of KAI1 in HT1376 bladder carcinoma cells, based on the results of the immunoblotting and transient gene expression analyses. 

Recent studies have indicated that the regulation of EMT markers is involved in the progression of bladder cancer [41–44]. However, the mechanisms involved in the EMT pathways in bladder cancer have not been defined conclusively. Our study also represents the first report demonstrating the IL6-regulated expression of the E-cadherin, N-cadherin, and vimentin protein in bladder carcinoma cells. A recent study indicated that MASPIN-positive cells were resistant to EGF-induced EMT, suggesting that MASPIN may be involved in the EMT pathways in urothelial-bladder carcinoma [45]. The expression of NDRG1 has been shown to be significantly correlated with the expression of E-cadherin in prostate cancer [46]. In addition, NDRG1 was observed to stabilize the E-cadherin protein through cellular-recycling pathways in prostate carcinoma cells [47]. The elucidation of whether IL6 directly or indirectly affects EMT markers in bladder carcinoma cells by inducing of MASPIN and NDRG1 warrants further investigation.

## 5. Conclusion

Our results indicate that the overexpression of IL6 upregulated the expression of the MASPIN, NDRG1, and KAI1 protein in HT1376 cells and that IL6 knockdown reduced the expression of these proteins in T24 cells. Our findings suggest the possible functions of IL6 in human bladder carcinoma cells. The dysregulation of the EMT and the altered MASPIN, NDRG1, and KAI1 gene expression induced by IL6 may lead to the modulation of tumorigenesis in bladder carcinoma cells.
